# An optical system to detect, surveil, and kill flying insect vectors of human and crop pathogens

**DOI:** 10.1038/s41598-024-57804-6

**Published:** 2024-04-08

**Authors:** Joseph M. Patt, Arty Makagon, Bryan Norton, Maclen Marvit, Phillip Rutschman, Matt Neligeorge, Jeremy Salesin

**Affiliations:** 1 United States Department of Agriculture, Agricultural Research Service, Fort Pierce, FL 34945 USA; 2 Global Health Labs (Formerly Global Good Fund I, LLC), Bellevue, WA 98007 USA

**Keywords:** Ecology, Zoology, Ecology, Diseases, Optics and photonics

## Abstract

Sustainable and effective means to control flying insect vectors are critically needed, especially with widespread insecticide resistance and global climate change. Understanding and controlling vectors requires accurate information about their movement and activity, which is often lacking. The Photonic Fence (PF) is an optical system that uses machine vision, infrared light, and lasers to identify, track, and interdict vectors in flight. The PF examines an insect’s outline, flight speed, and other flight parameters and if these match those of a targeted vector species, then a low-power, retina-safe laser kills it. We report on proof-of-concept tests of a large, field-sized PF (30 mL × 3 mH) conducted with *Aedes aegypti*, a mosquito that transmits dangerous arboviruses, and *Diaphorina citri*, a psyllid which transmits the fatal huanglongbing disease of citrus. In tests with the laser engaged, < 1% and 3% of *A*. *aegypti* and *D*. *citri*, respectfully, were recovered versus a 38% and 19% recovery when the lacer was silenced. The PF tracked, but did not intercept the orchid bee, *Euglossa dilemma*. The system effectively intercepted flying vectors, but not bees, at a distance of 30 m, heralding the use of photonic energy, rather than chemicals, to control flying vectors.

## Introduction

Flying insects that transmit human, animal, and plant pathogens have global impacts on human health, agriculture, and ecological stability^1,2^. Eighty percent of the world’s population is vulnerable to vector-borne diseases, which account for ca.17% of all infectious diseases and kill over 700,000 people per year, primarily children^2^. For example, malaria, a parasitic infection transmitted by *Anopheles* mosquitoes, infected 249 million people worldwide in 2016 and caused over 600,000 deaths, mostly young children^2,3^. Dengue, which causes 40,000 deaths per year^2^, is transmitted by *Aedes* mosquitoes as are other arboviruses, such as chikungunya fever, Zika Virus, West Nile Virus, and yellow fever^2,4^. The parasite that causes leishmaniasis is transmitted by female phlebotomine sandflies, which like mosquitoes, feed on blood to produce eggs^5^. It is estimated that 700,000 to 1 million new cases of leishmaniasis occur annually, primarily among the world’s poorest people^6^. The health of livestock, domestic animals and wildlife is also seriously affected by vector borne diseases, such as trypanosomiasis, Rift Valley Fever, and blue tongue virus^7–10^. With rapid globalization, ecological links within a native pathosystem can be replaced in a naïve area with new vectors and hosts, exacerbating efforts to limit disease spread^11–13^. Likewise, crop production suffers worldwide from plant diseases spread by flying insect vectors such as aphids, whiteflies, planthoppers, and psyllids^14–21^.

Control of insect vectors relies primarily on chemical insecticides, which are often the only practical control solution^22–24^. However, reliance on insecticides is costly and it negatively impacts the health of humans, non-target organisms, and the environment^22,25–28^. Alarmingly, most vector species are already resistant to available insecticides^29–37^, and insecticide resistance has now been reported in all major malaria vector species and against all classes of insecticide^38–40^.

Increasing global temperatures increases insect vector abundance and distribution, bringing outbreaks of vector-borne diseases to new areas, exacerbating control efforts in at-risk areas^41–56^, and promoting more insecticide use^57–59^. At higher temperatures and CO_2_ levels, insecticides decompose more quickly, and insects detoxify them more rapidly, which may decrease their efficacy as the climate warms^60–63^. Moreover, synergistic interactions between rising temperatures and increasing insecticide applications can aggravate insect pest outbreaks^64,65^. As the climate warms, the rapid evolution of vector resistance to available insecticides will be promoted by factors such as increases in vector activity levels, movement patterns, and generations per year^33,42,51,66–69^. There is also the troubling occurrence of increased vector resistance to pyrethroids, which are used to treat bed nets, in vector populations inhabiting agricultural areas where pyrethrins are used against crop pests, making it more difficult to control them with these materials in inhabited areas^70–74^. Developing and managing vector resistance programs to extend the life of available insecticides is among the most difficult and important challenges facing global public health and food security in the near term, especially as the climate warms and changes^36,75–79^.

The capability to accurately monitor the abundance and distribution of flying insect vectors within a target area is critical to implementing regional management plans aimed at reducing vector populations, managing pesticide resistance, and eliminating pathogen transmission^80,81^. Providing accurate estimates of vector abundance and distribution within a given area is contingent on obtaining reliable counts of vectors at light traps, CO_2_ traps, or other trapping devices^82–85^. However, efficiency of traps with respect to delivering reliable captures depends on several factors and trapping may not always provide accurate estimates of vector population size^86–88^. Still, examination of specimens caught in traps is the best, and sometimes only, way to identify and count individuals of a particular vector species. Retrieval of specimens from traps is required for genetic, morphological and biochemical analyses and examination, which are necessary steps for conducting fundamental studies on insecticide resistance in vector populations and for developing and implementing insecticide resistance management strategies^33^. Recent advances in sensor and computer vision, machine learning, and the Internet of Things technology have greatly improved the functionality and effectiveness of insect traps with respect to their capability to identify and classify vectors and to perform these tasks autonomously^89–96^. However, most insect traps in use today have the drawback of being labor- and time intensive to set-up, retrieve, and analyze, are difficult to use in unwelcoming terrain or over extensive sampling areas, and are subject to weather conditions and events^97,98^. This makes it difficult in many scenarios to accurately assess vector populations without incurring high costs^84,99,100^. In addition, traps sometimes fail to adequately sample important or infected vectors, which can lead to errors caused by sampling bias and misinterpretation of the data^101^. More effective vector monitoring and control approaches are urgently required to address insecticide resistance and prevent vector-borne diseases from increasing and spreading^74,102^.

An emerging technology for monitoring insect vectors is the use of electromagnetic radiation coupled with advanced optical imaging systems, signal processing technology, computer vision, and machine learning^92,103–110^. Advances in entomological radar and lidar have permitted successful survey and monitoring activities of insect fauna at high- to mid-altitudes^111–113^. This technology has provided important insights into insect migration and diurnal and seasonal flight differences as well as revealed the ecological and agricultural importance of insects inhabiting different elevational or altitudinal niches^114,115^. The introduction of harmonic radar, in which an insect is fitted with a transponder energized by the radar, permitted the tracking of individual insects at ground level^116–118^ or even using UAV drones to enhance tracking ability^119^. Near ground lidar tracking is now sufficiently sensitive to perform fine-scale observations such as the recording the anomalous daytime flight activity of mosquitoes during solar eclipses^120^ and quantifying insect pest abundance above rice paddies under different meteorological conditions^121^. Recently, a high resolution lidar scanning a 500 m transect outside a rural African village was able to quantify the daily dispersal of mosquitoes in a village and revealed that the distribution of each gender differed in relation to the village^122^. Over the course of that study, a quarter of a million insect observations were made, and several insect taxa were identified based on their wingbeat modulation signatures.

Infrared laser-based systems coupled with machine learning are now sensitive enough to determine the sex of individual mosquitoes in flight^123,124^. Because male and female mosquitoes produce different frequencies while in flight, the amplitude of the backscattered light received from each gender differs and the signal can be used as a basis for gender identification and discrimination. Sufficient image resolution permitted such systems to distinguish gravid from non-gravid female mosquitoes^125^. An infrared laser-based optical sensor performed continuous surveillance of flying insects for 80 days^126^. The device recorded light backscattered by insects transiting through its laser beam and compiled information about each insect’s wing beat frequency, transit time across the beam, and the ratio of its wings to body dimensions to identify the insects to specific taxonomic groups. Data from this survey enabled the determination of the aerial densities, circadian rhythms and peak activities of each taxonomic cluster of insects. These results demonstrate that optical sensors, coupled with machine learning, are a viable methodology for performing non-intrusive, automatic, measurements of insect population dynamics over extended periods of time.

Just as innovations in optical and machine learning systems are making tangible progress towards permitting real time detection, identification, and tracking of flying insects, combining this technology with photonics is providing the means to intercept and kill them^127–137^. Studies have demonstrated that lasers pulses can kill insects from as large as a roach (*Blatella germanica*)^136^ to as small as a whitefly (*Bemisia tabaci*)^137^. In lab tests, laser pulses were shown to be capable of killing flying mosquitoes (*Anopheles stephensi*, *Culex pipiens*), Asian citrus psyllid (*Diaphorina citri*) and, spotted wing drospophila (*Drosophila suzukii*)^127–131^. Aphids, which are among the most important vectors of plant viruses, are vulnerable to laser pulses while they feed on plants^134,135^. In this system, a robot bearing the photonic system moves down a row of crop plants, interrogates each plant, and pulses a laser to individual aphids attached to the stems and leaves.

**Figure 1:**
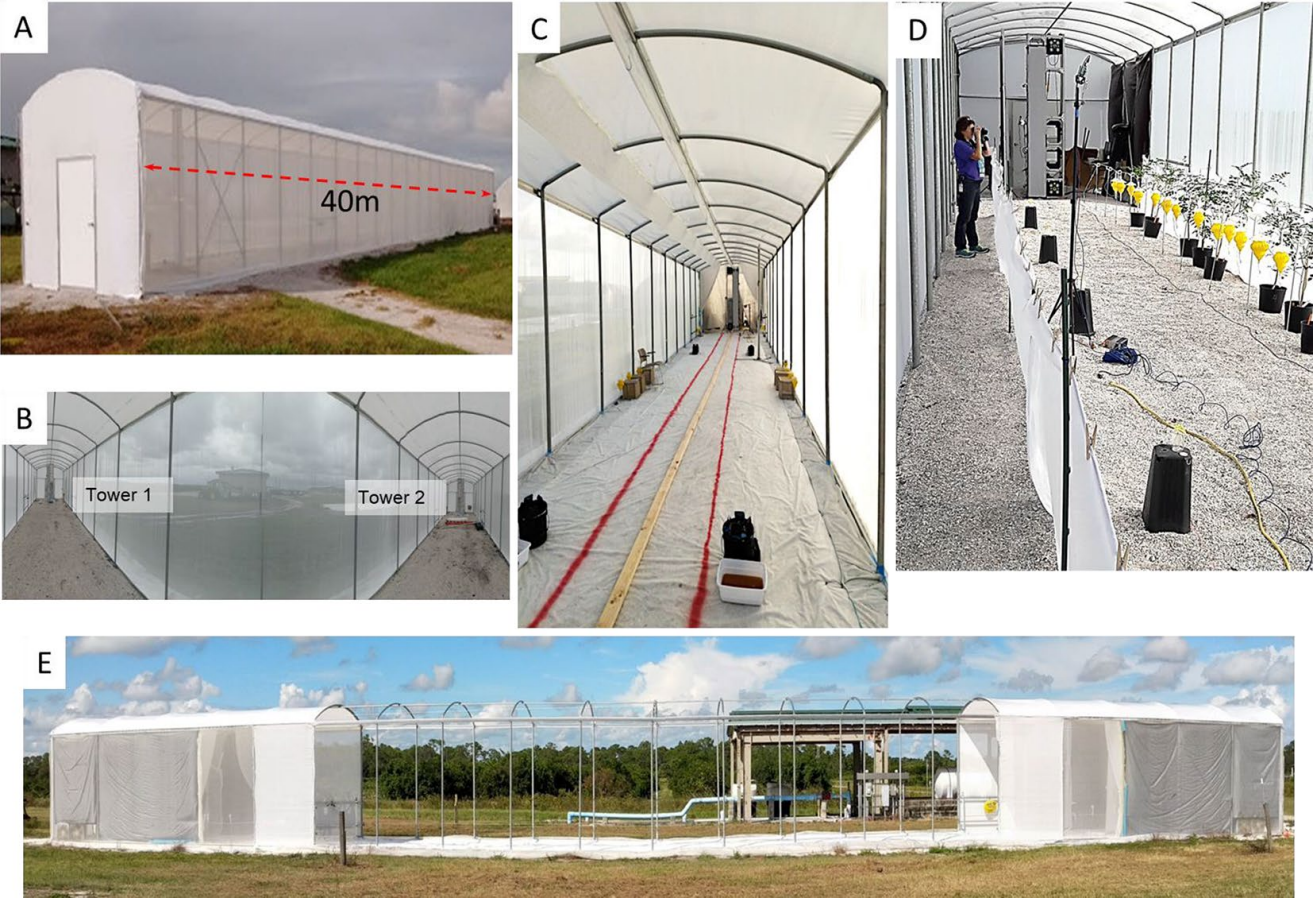
The screenhouse (40 m L × 3.05 m W × 3.66 m H) built to test the Photonic Fence at the USDA-Agricultural Research Service citrus grove in Fort Pierce, FL. (**A**) Exterior view. (**B**) Wide angle view of the interior showing the placement of Photonic Fence towers at both ends of the screenhouse. (**C**) Interior view from one Photonic Fence tower to the other showing the placement of traps for *A*. *aegypti* recovery. (**D**) View of yellow 3D traps arranged along recovery side of screenhouse during preliminary recovery tests. Note that the observer is located on the vector release side of the screenhouse and that the psyllids must fly across the active zone to reach the traps and potted host plants set up on the opposite side. See Fig. 10B for the actual set-up used during tests. (**E**) Screen house set-up for the open-air test to evaluate the Photonic Fence’s capability to track and target ambient levels of insects flying during dusk.

**Figure 2:**
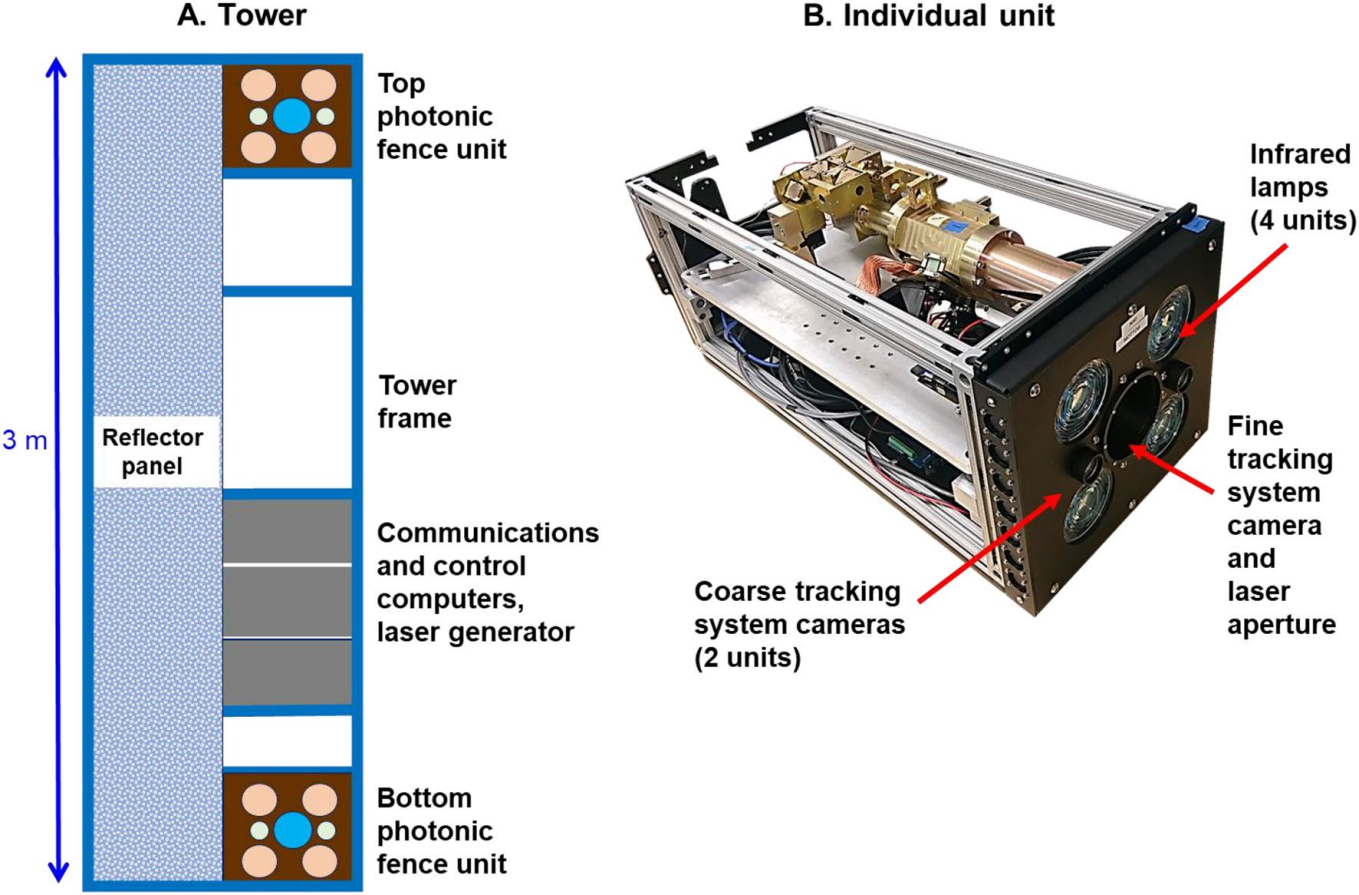
Diagrammatic representation of the Photonic Fence system: (**A**) Front view of the support tower showing the position of the tower frame, individual Photonic Fence units, reflector panel, communications and control computers, and laser generator. (**B**) Isometric view of an individual Photonic Fence unit showing position of infrared lamps, fine tracking system and laser aperture, and coarse tracking system apertures.

**Figure 3:**
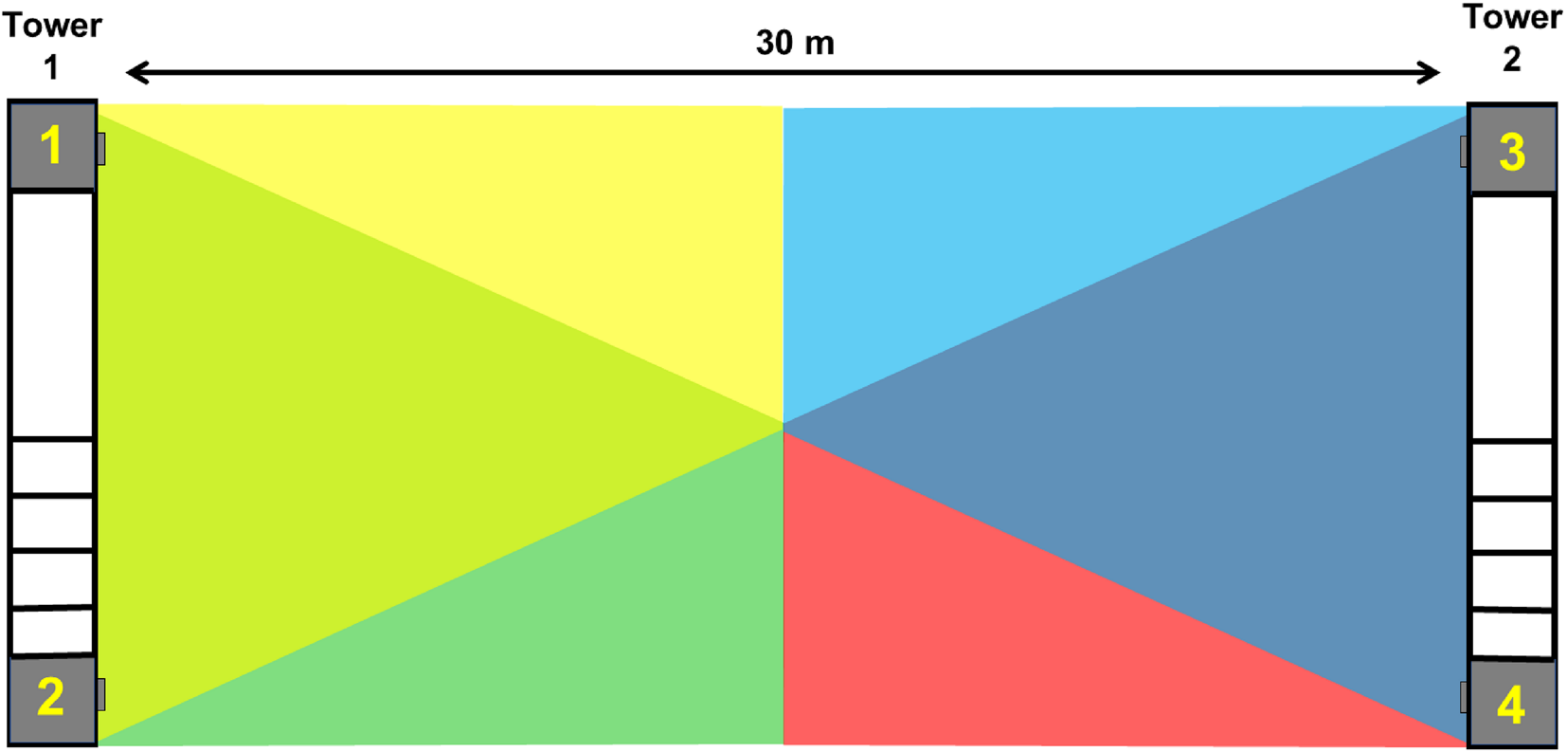
Diagrammatic representation of showing the placement of the Photonic Fence towers and the overlapping photonic fields generated by each of the four Photonic Fence units.

Here we report on the first tests of a field-scale sized all-optical system, the Photonic Fence, which is designed to identify and intercept small flying insect vectors over a wide area^127–129^ (Fig. 1). The Photonic Fence units, developed by Global Health Labs (formerly Global Good Fund), are mounted on two towers placed opposite of each other (Figs. 1, 2 and 3). Each unit is comprised of three independent modules which function sequentially. One module detects insect motion, and the others tracks and identifies the targeted insect. The initial detection of an insect is achieved by acquiring a video image of it when it crosses the virtual boundary of the system, which is referred to as the ‘active zone’. The active zone consists of a plane of infrared light (Fig. 3) projected by infrared lamps mounted on one Photonic Fence tower and reflected from a retroreflector on the other tower which is placed opposite of it at a defined distance (Fig. 3). Insects traversing the active zone are back illuminated by the infrared light reflected from the retroreflectors and thereby cast silhouettes. The first module, the ‘coarse tracking’ system (Fig. 2B), uses a pair of stereoscopic cameras to image the silhouettes and machine vision algorithms to provide real-time reconstruction of each insect’s silhouette and identify its three-dimensional (xyz) location within the active zone. If the insect’s size and speed fall within a pre-defined range, it is further interrogated by the second module, the ‘fine tracking’ system (Fig. 2B). This module uses a high-speed camera and a fast-scanning mirror to keep the targeted insect in the middle of the camera’s field of view. By comparing the target’s size, speed, and other parameters to those of vector species contained in a database, the system identifies the targeted insect. If the insect is a vector, then the third module (Fig. 2B) can direct a very brief pulse of a low-power, retina-safe laser to kill it. Sophisticated software integrates the module components, providing real-time, continuous coverage of the active zone. The laser system is designed so it will not fire if people or other animals enter the active zone^127–129^.

During development of the Photonic Fence, the capabilities of multiple types of lasers to kill anaesthetized mosquitoes (*Anopheles stephansi*) were evaluated and the primary optical parameters involved in causing thermal damage to internal organs and subsequent morbidity or mortality were identified^127^. Besides the laser’s wavelength (nm), these included beam (spot) diameter (mm), pulse duration (ms), and pulse energy (mJ). The tests demonstrated that anaesthetized mosquitoes could be killed by laser pulses to the thorax at various wavelengths, beam diameters, durations, and power conditions. Green and far-infrared wavelengths were found to be more effective in incapacitating mosquitoes than near- and mid-infrared wavelengths.

Follow-up studies were performed with vectors flying in small acrylic enclosures positioned in the active zone to evaluate and optimize the Photonic Fence’s tracking system and its integration with the laser system^128,129^. During tests with flying mosquitoes (*A. stephansi*), the system successfully derived individual mosquitoes’ velocity, and linear and angular acceleration before, during, and after the laser pulse from its xyz position, which was continuously monitored by the tracking system^128^. Spatial positioning of the mosquitoes was obtained by assigning each tracked mosquito a coordinate in image space based on its image centroid, from which the actual azimuth and elevation relative to the optical center of the camera could be calculated. To evaluate interception performance during the laser pulse, the fine tracking system was used to determine how much of the laser’s 2.5 mm beam diameter overlapped with the mosquito’s thorax and abdomen It was found that a tracking error of < 2 pixels for a minimum of 50 ms was required for the system to accurately target and hit the mosquito with the directed laser. The initial tests of this laboratory study were performed with the acrylic enclosures positioned 2 m from the Photonic Fence unit. After further calibration, the Photonic Fence was capable of targeting and killing all the mosquitoes (in this case both *A. aegypti* and *C. pipiens*) flying in an enclosure placed 30 m from it.

The Photonic Fence installation described in the current study had a large active zone measuring 30 m long × 3 m high × 0.3 m wide (Figs. 1 and 3). It was the largest and most complex design of the Photonic Fence to date and integrated the sub-systems that were tested and optimized previously with respect to laser control and delivery, target tracking, and target selection^127–129^ This version of the Photonic Fence was designed to maximize performance at the expense of size, weight, cost, and other system variables, with the expectation that those variables would be optimized during a later productization stage. The objective of the study was to determine if the Photonic Fence could detect, track, and intercept vectors across the entirety of an active zone of a size envisioned for deployment in the field.

The yellow fever mosquito, *Aedes aegypti* (L), was chosen as the representative human pathogen vector for this study because it and its congener *Aedes albopictus* (L), vector dangerous arboviruses, such as yellow fever, dengue, chikungunya, and Zika^138–140^. Once a forest-dwelling zoophilic mosquito, *A*. *aegypti* gradually adapted to changing weather and environmental conditions and became an anthropophilic, urban-dwelling species^137–141^. The females prefer to oviposit in containers holding stagnant water, such as flowerpots, discarded containers, tires, etc. and rest in habitations^142^. *A. aegypti* is 4–7 mm long and may fly over 100 m while searching for hosts and oviposition sites^143,144^. These adaptions have facilitated the spread and establishment of *A*. *aegypt*i in new areas inhabited by immunologically naive human populations and led to a shift in the epidemiology of these viral diseases^136–139^. A further complication is that these mosquitoes can disseminate multiple viruses simultaneously^145,146^, which further shows that more effective means to surveil and control *Aedes* mosquitoes are needed, especially when managing arbovirus outbreaks^147^.

The Asian citrus psyllid, *D. citri* (Kuwayama), was chosen as a representative vector of a serious crop pathogen. This psyllid is an obligate phloem feeder of *Citrus* and closely related plants in the Rutaceae family. It vectors *Candidatus* Liberibacter asiaticus, a gram negative, phloem-limited bacterium that is the putative causal agent of huanglongbing disease of citrus^148–150^. Huanglongbing is the most devastating disease of citrus in the world: infected trees initially have mottled, misshaped leaves and green, bitter fruit and they typically die a few years later as there is currently no therapeutic treatment available against the pathogen^151,152^. Huanglongbing has seriously impacted major citrus-producing regions globally^153–156^. For example, citrus production in Florida, the largest orange juice producing state in the U.S., has decreased by over 70% since 2005, when the disease was first detected there^153,156,157^. Adult *D*. *citri* are typically 2.7–3.3 mm long^158^ and can disperse widely across landscapes^159–161^. Growers rely heavily on insecticides to control *D*. citri^162^, which consequently has resulted in the emergence of insecticide-resistant psyllid populations^163–167^. Because there are limited therapeutic treatments available for infected citrus trees^168,169^, new control measures are needed for *D*. *citri*. An earlier study demonstrated that the laser system of the Photonic Fenced could kill anaesthetized *D. citri*^129^. Dosing measurement showed that psyllid mortality occurred at a low energy level of 2 mJ, and that 90% mortality was reached at 15 mJ from pulses of a continuous wave 445 nm laser that lasted from 3 to 28 ms. The detection and tracking modules could be re-programed to track *D*. *citri* flying in a transparent acrylic chamber. Further testing of the integrated system demonstrated that it could detect, track and intercept *D*. *citri* at close range^128^. Other researchers have also shown that *D*. *citri* could be killed by laser pulses^130^.

**Figure 4:**
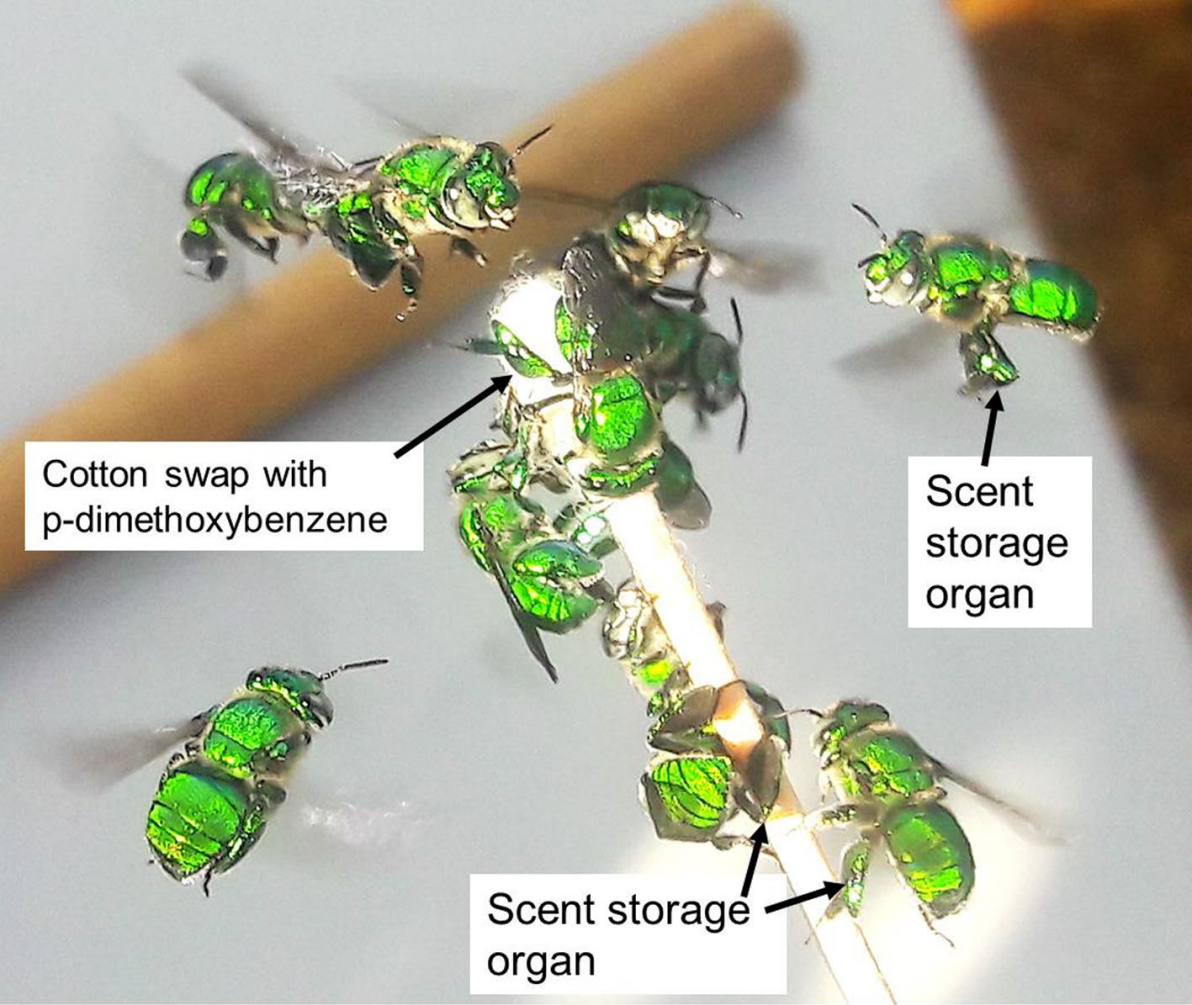
Photo of males of the orchid bee, *E. dilemma*, which were used as a representative non-target species. The males were attracted to a cotton swab which was dipped into a scent bait, p-methoxybenzene. The males collect the scent, store it in a specialized organ located on their rear legs, and then use it to produce a courtship pheromone. Photo by J. M. Patt.

The orchid bee, *Euglossa dilemma* (Friese), a Central American euglossine bee species established in southern Florida^170,171^, were used to test the Photonic Fence’s capability to discriminate between target and non-target insects. While they are exotic to Florida, euglossine bees are critical pollinators in many tropical plant communities in their native range^172^ and play a highly beneficial role in those regions. Males of this orchid bee were used as a representative non-target species because they don’t sting, making them easy to handle, are ca. 1.3 mm long, which is close in size to the honey bee (*Apis mellifera* L.)^171^, and are abundant in the test locality. Male orchid bees have a strong co-evolutionary relationship with certain orchid species, are highly attracted to certain floral volatiles^173–175^; and can be readily collected at scent baits^176^ (Fig. 4).

## Results

### Initial mosquito tracking and interception tests

**Figure 5:**
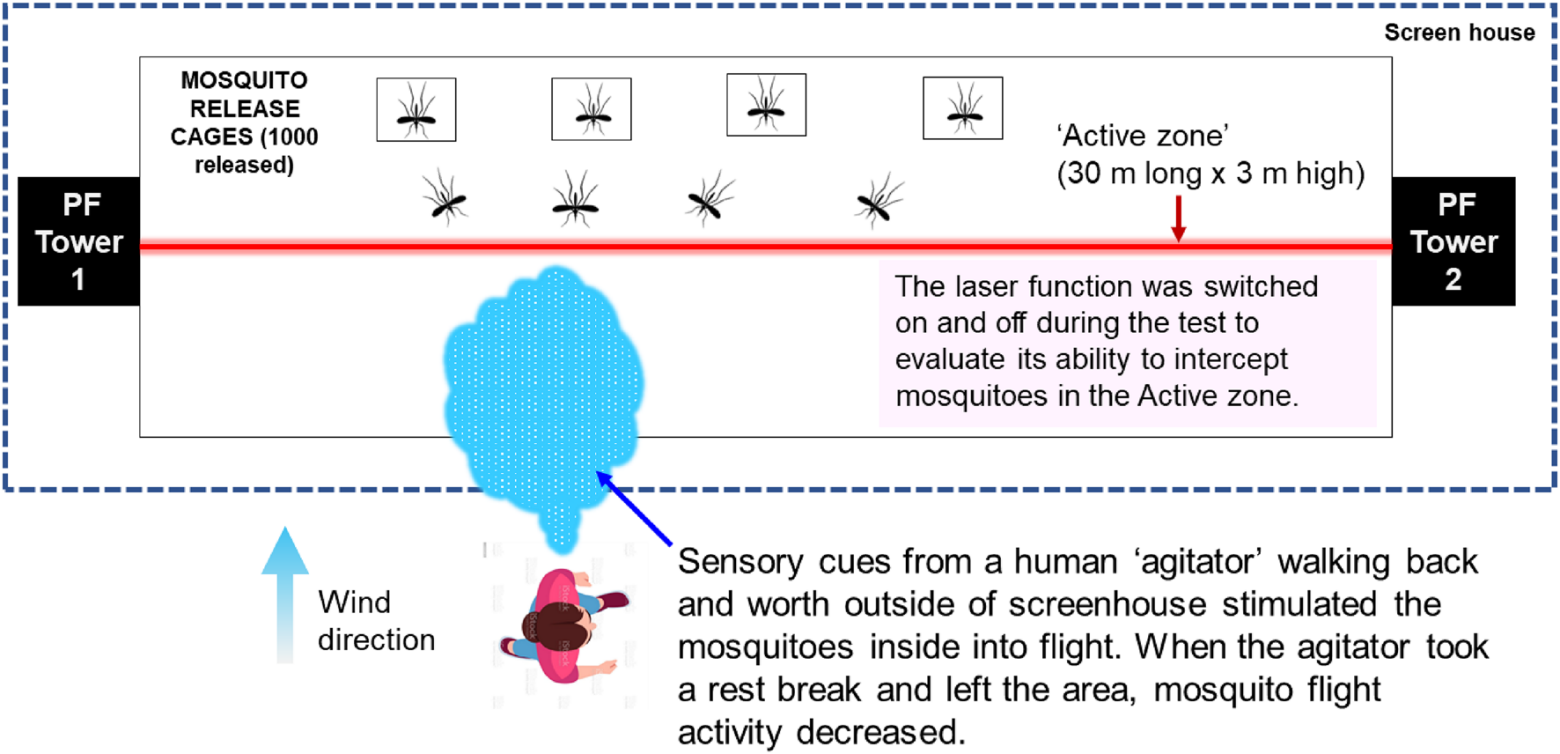
Diagrammatic representation of the set-up for the initial 5.5-h long test of the Photonic Fence. A human ‘agitator’ walked along the outside of the screenhouse to help stimulate mosquito (*A*. *aegypti*) activity. Initially, the laser was disengaged, and the system measured the flight activity level of the mosquito population when the agitator was next to the screenhouse versus when they tool a rest break and were away from it. Towards the end of the test, the laser was engaged to test its effectiveness in reducing the mosquito population in the screenhouse.

**Figure 6:**
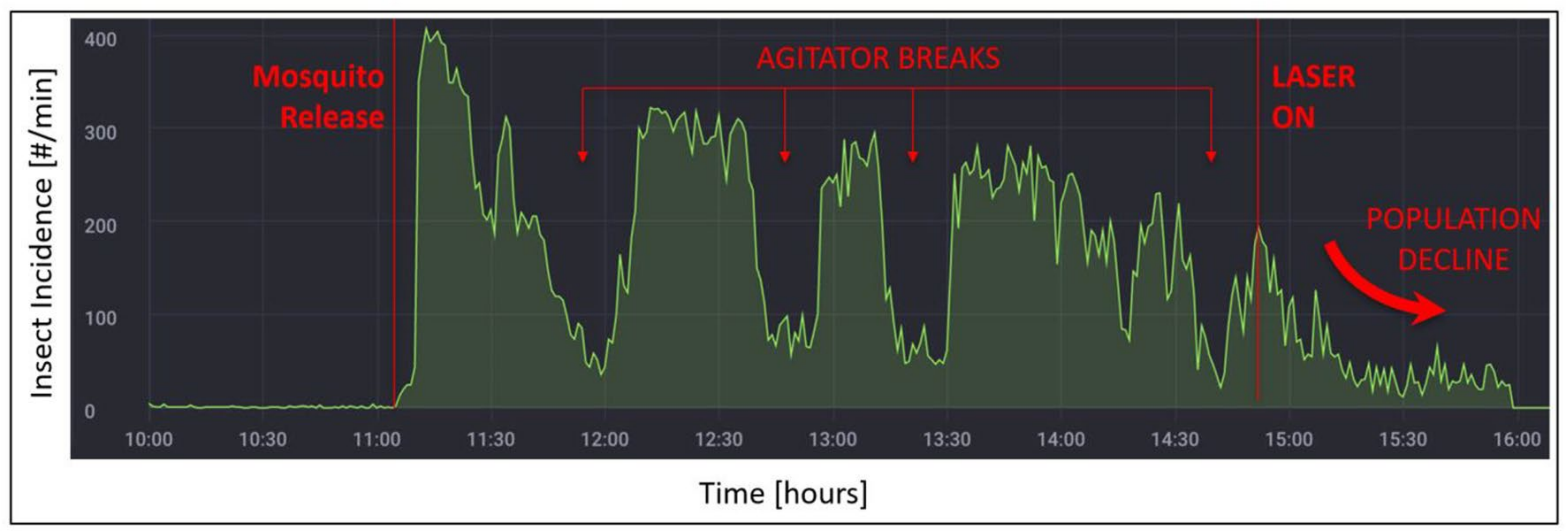
The number of detections of flying *A*. *aegyti* over the 5.5 h-long initial test of the Photonic Fence. For this test, 1000 *A*. *aegypti* were released inside the test area. During the initial 4 honly the monitoring mode was active. Note that when the human agitator took a break and walked away from the screen house, mosquito activity declined. During the last hour of the test, the lethal laser mode was engaged, and the population of mosquitoes declined in the screen house.

**Figure 7:**
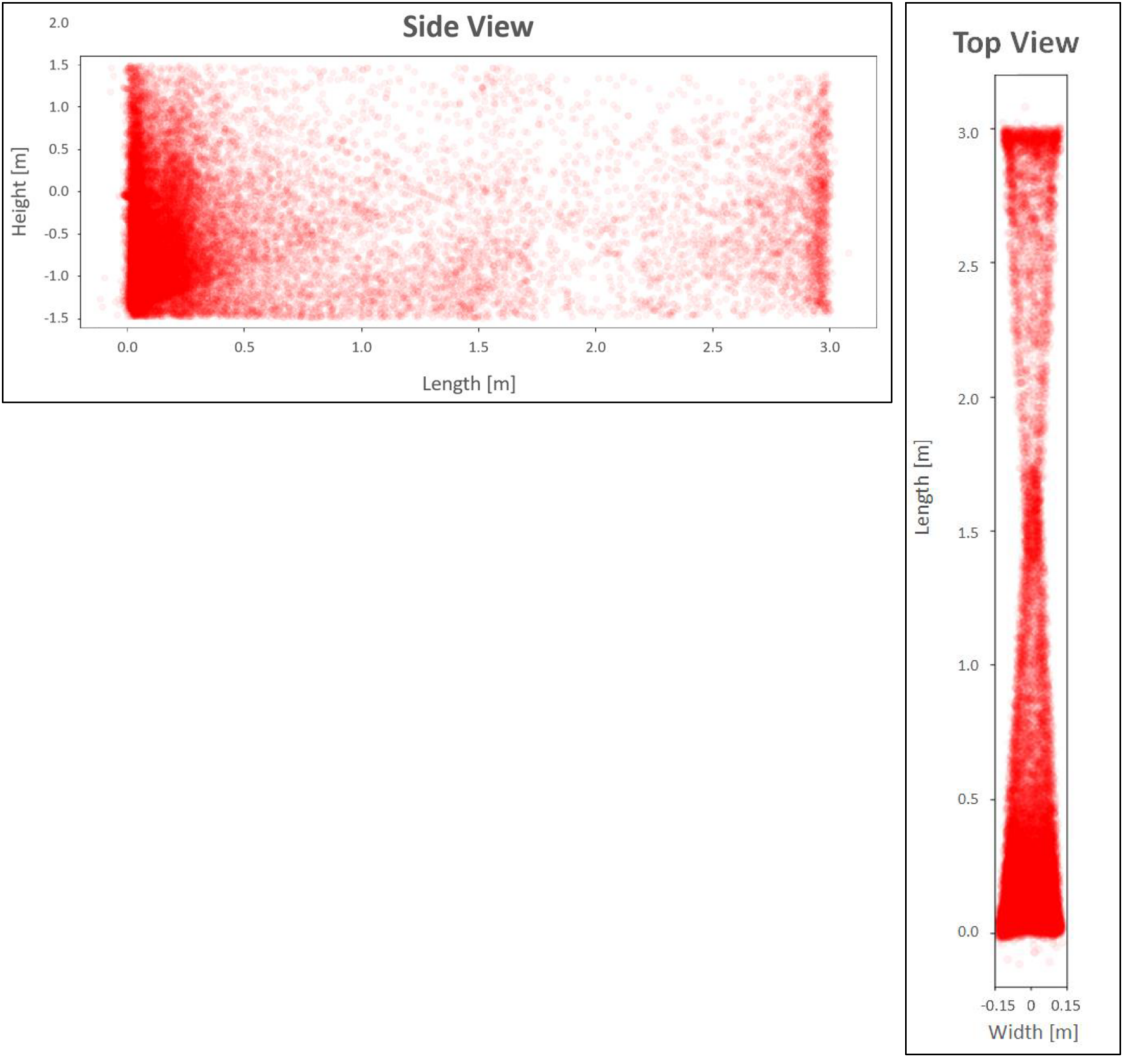
Cumulative tracking events of 1000 *A. aegypti* over the course of the initial test, showing views from the side and top of the active zone. Each red dot was a tracking event and their distribution shows that the mosquitoes were successfully tracked throughout the active zone of the Photonic Fence.

**Figure 8:**
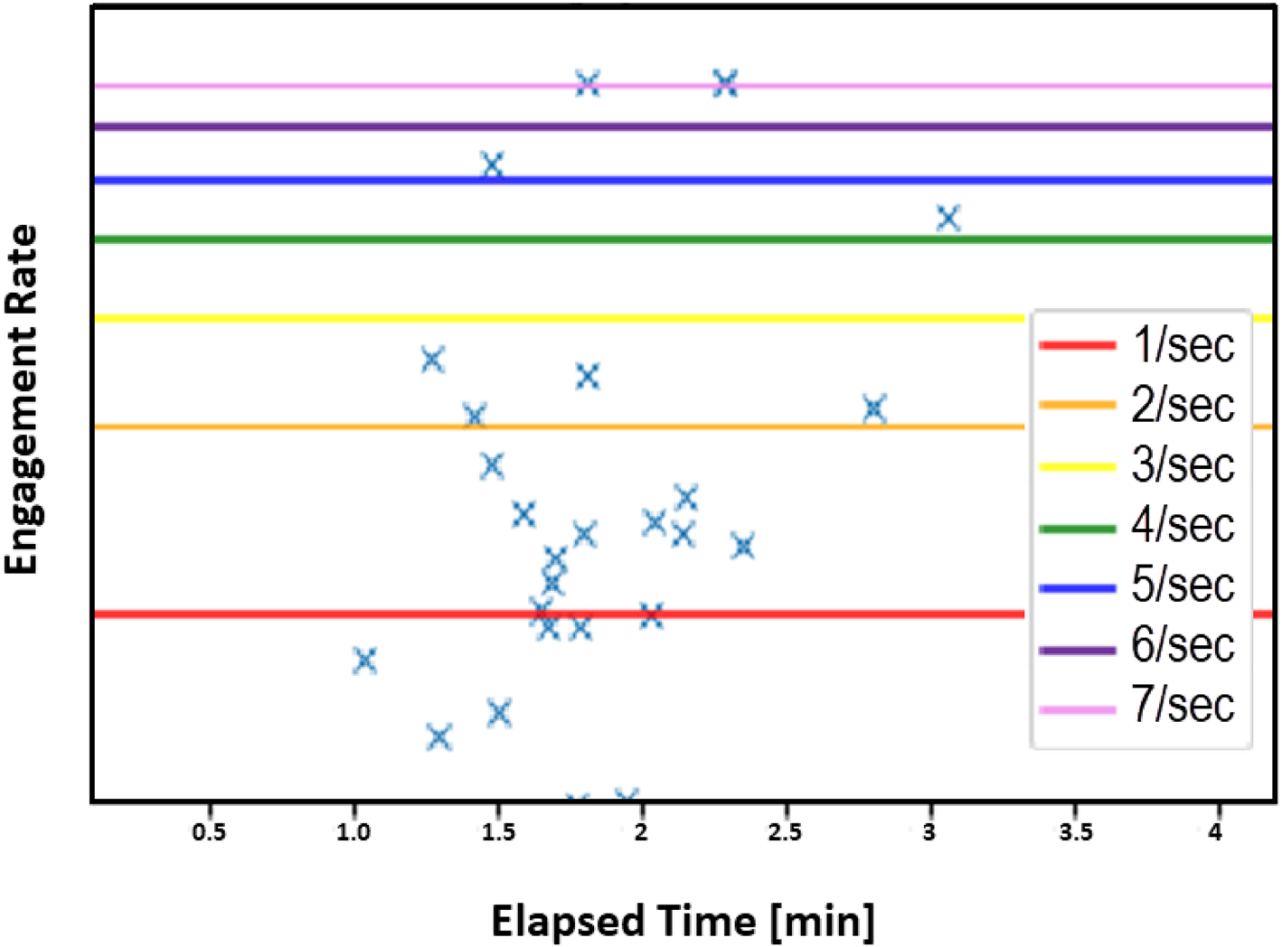
Lethal laser engagement rate with *A. aegypti* during the initial test of the Photonic Fence showing that the system could intercept up to 7 mosquitoes per second.

During the first 4 h of an initial mosquito tracking and interception test, the detection, interrogation, and tracking modes were active while the laser function was silenced. Following the release of 1000 *A*. *aegypti* in the screenhouse, with a human ‘agitator’ walking back and forth on the upwind side of the screenhouse exterior, a peak of just over 400 mosquitoes per minute were detected in the active space extending along the middle of the screenhouse (Fig. 5). At the 45-min point, the agitator took a break and left the vicinity of the screenhouse, and the number of mosquitoes detected per minute decreased to around 50 individuals per minute. When the agitator returned, the detection level again rose to greater than 300 individuals per minute. The agitator took two more breaks over the next 2 h, and each time the number of mosquitoes detected decreased to between 50 and 100 individuals per minute. The numbers then rebounded to between 200 and 280 individuals when the agitator returned. Four hours after the start of the test, the laser mode was engaged. During the final 60 min of the test the mosquito population within the screenhouse declined from laser-inflicted mortality and their detection levels dropped steadily to 50 or fewer individuals per minute (Fig. 6). A post-hoc analysis of the system’s responses showed that the mosquitoes were detected within the entire 180 square meter active space (Fig. 7). The laser typically engaged 1–2 mosquitoes per second but could engage up to 7 targets per second (Fig. 8).

### Flying vector interception efficiency tests

#### Aedes aegypti

**Figure 9:**
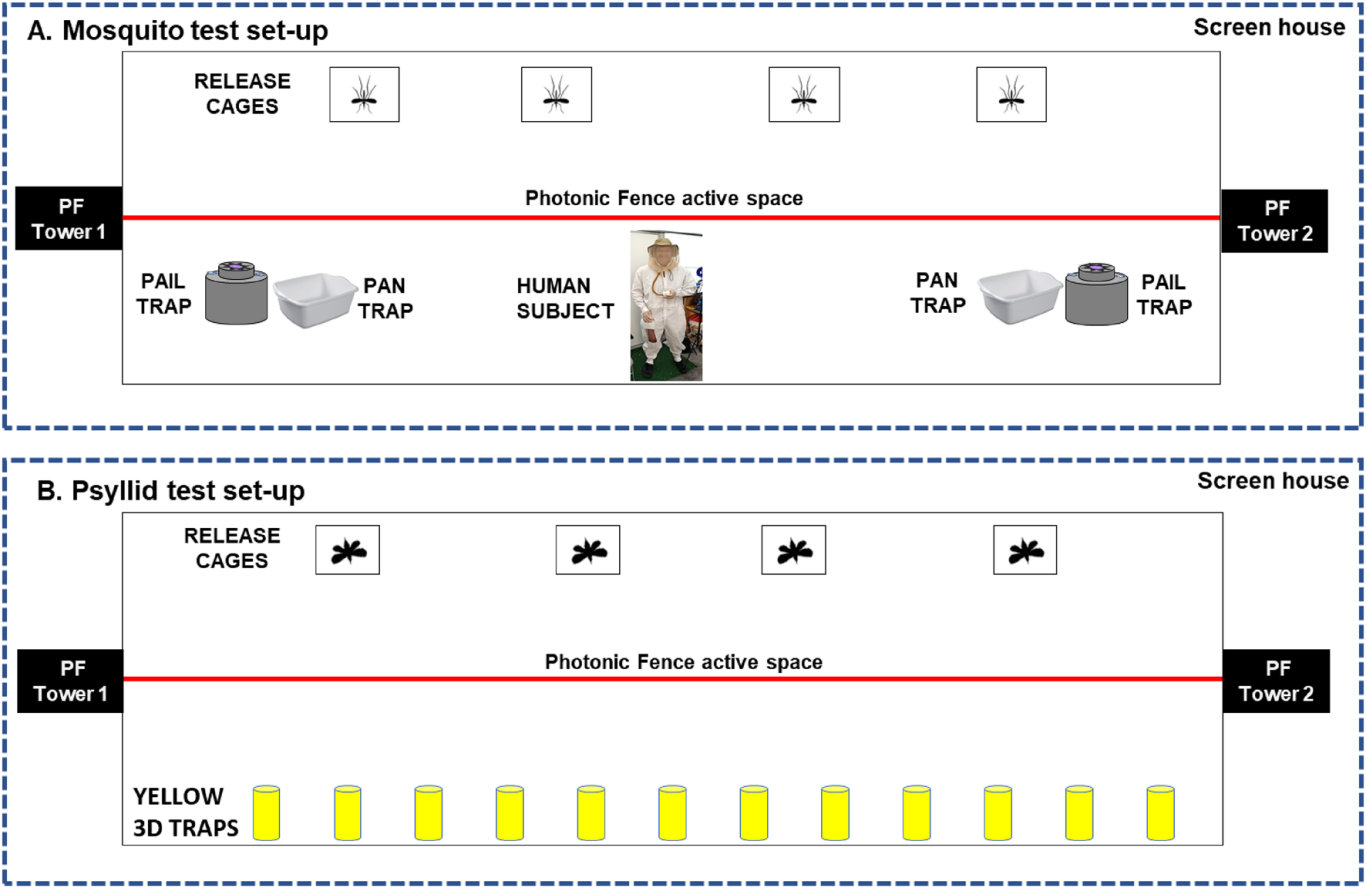
Diagrammatic representation of the set-up for testing the efficiency of the system’s ability to intercept flying insect vectors. Comparisons were made of vector recovery while only the monitoring mode was activated versus that with both the monitoring mode and laser activated. (**A**) Tests with mosquitoes, showing relative positions of cages for releasing *A*. *aegypti*, two pail traps with visual and olfactory attractants, two pan traps with seasoned water, and a human subject covered with a bee net and white overalls equipped with a 100 cm^2^ opening which exposed the human subject’s skin while seated during the test. (**B**) Tests with psyllids, showing relative positions of cages for releasing *D*. *citri* and yellow 3-D traps.

**Figure 10:**
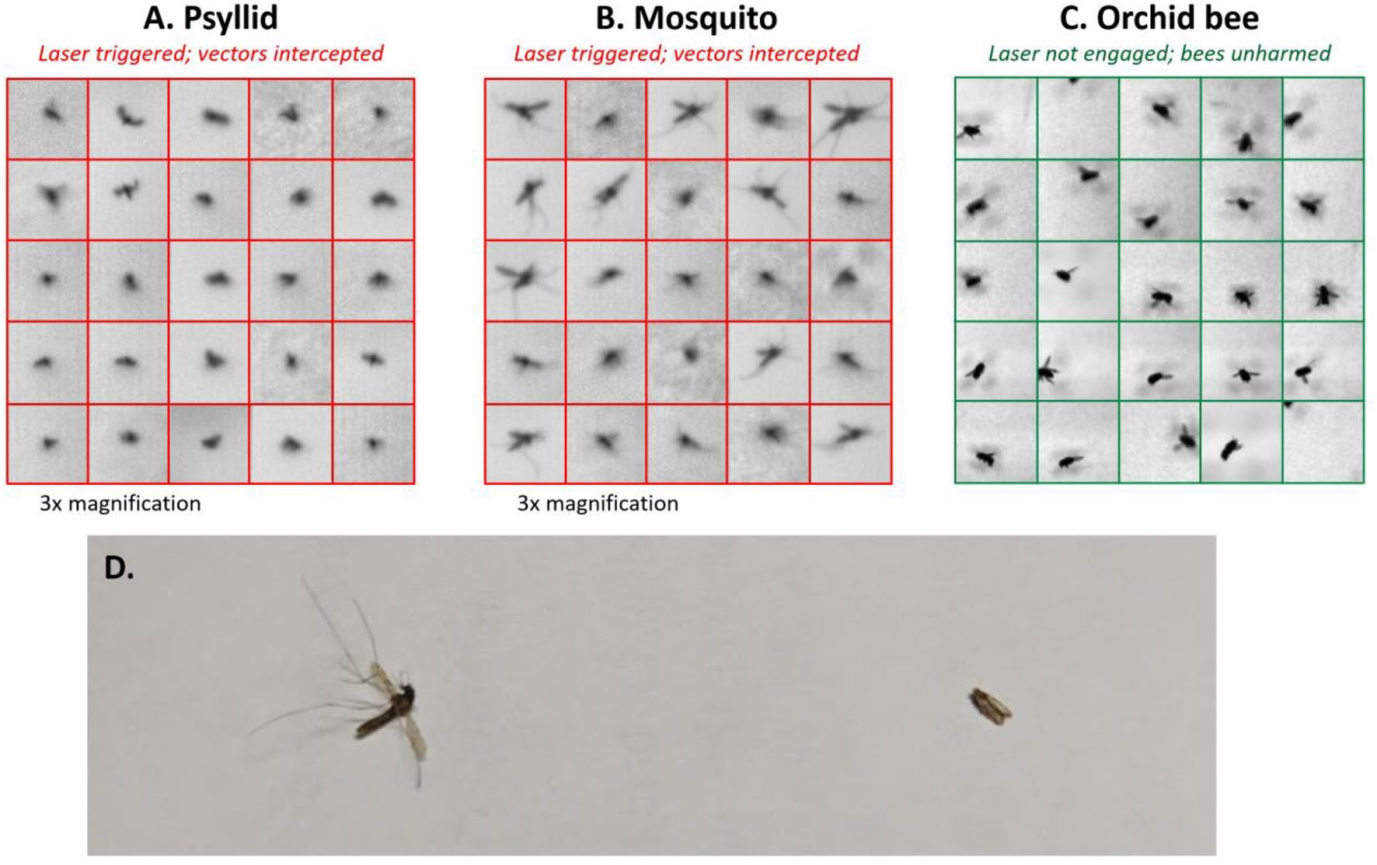
Results of the target discrimination tests in which there was a 100% laser pulse triggering when the pathogen vectors *A. aegypti* and *Diaphorina citri* were detected versus 0% laser pulse triggering when the pollinator *E. dilemma* was detected. Shown are silhouette images captured by the fine tracking system of the Photonic Fence of individual: (**A**) Yellow fever mosquitoes (*A*. *aegypti*), (**B**) Asian citrus psyllid (*D*. *citri*) and **C.** orchid bees (*E*. *dilemma*) in flight. Images show the last fine tracking frame prior to the target validation algorithm correctly setting a do-not-engage status. (**D**) Photo showing side-by-side comparison of mosquito and psyllid intercepted and killed by a laser pulse. Note the overall difference in the dimensions of each vector species.

In a release and recovery test (Fig. 9A) with only the monitoring mode active, 38.3% of the 350 *A*. *aegypti* released at the beginning of the test on one side of the Photonic Fence were recaptured either on a human subject (61 total) or in the mosquito traps (73 total) 60 min following their release (Table 1; Fig. 10A). In the subsequent test with both the monitoring and laser modes active, only 3 (0.86%) of the 350 released mosquitoes were recaptured on the human subject while none were recaptured in the mosquito traps.

**Table 1:** Results of tests comparing the recovery of flying vectors with only the monitoring mode active versus with both the monitoring and laser modes active. Each test ran for 60 min. **A.** Recovery of *A. aegypti* mosquitoes on a human subject and in traps. **B.** Recovery of Asian citrus psyllid (*D*. *citri*) on yellow 3-D traps.

**Recovery point:**	Only monitoring mode active	Both monitoring + laser modes active
**A.** Yellow fever mosquito (*Aedes aegypti*) (Single test performed in each mode, with 350 mosquitoes released)		
Number of mosquitoes settled on skin patch of human subject	61	3
Combined numbers of mosquitoes collected in pan and pail traps	73	0
**% Recovered**	**38.3**	**0.86**

Test number	Monitoring mode only (Total recovered and **% recovered**)	Both monitoring + laser modes active (Total recovered and **% recovered**)
**B.** Asian citrus psyllid (*Diaphorina citri*) (600 psyllids released per test. A single test was performed with only the monitoring mode active and 2 tests were performed with both the monitoring and laser modes active.)		
1	112 (**18.7%**)	NA
2	NA	22 (**3.7%**)
3	NA	14 (**2.3%**)

#### Diaphorina citri

In the initial release and recovery test (Fig. 9B) with only the monitoring mode active, 18.7% (112 total) of the 600 *D*. *citri* released at the beginning of the test on one side of the Photonic Fence were recaptured in the 3-D yellow traps. In the following two tests with both the monitoring and laser modes active, 3.7% (22) and 2.3% (14) of the 600 released psyllids were recaptured in the 3-D yellow traps (Table 1; Fig. 10B).

### Target discrimination test with bees

All 10 individual male orchid bees (*E*. *dilemma*) released in the screenhouse were recovered alive following a 60-min test period with both the monitoring mode and laser engaged (Fig. 10C).

### Open-air test

**Figure 11:**
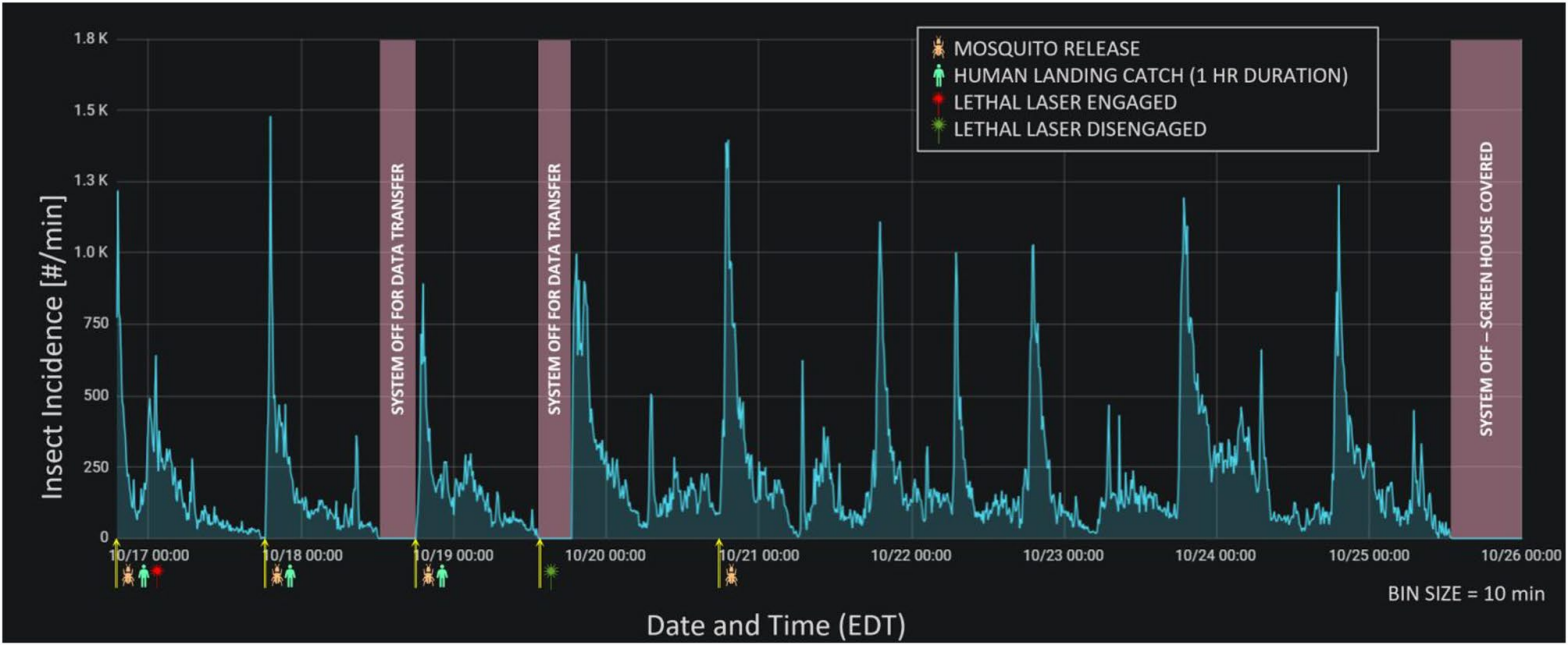
Results of 10-day long open-air test with the middle section of screening removed from the screen house as shown in Fig. 1E. During the initial 96-h period, three releases were made in the screenhouse of 10,000 *A*. *aegypti* at the dusk, the peak period of daily ambient flying activity at the study site. The laser was engaged from the start of the test until 72 h had elapsed. After the laser was disengaged, a control test was conducted at dusk on day 5 in which 10,000 mosquitoes were released but the laser continued to be disengaged. During the last 4 days of the test, no further releases were conducted while the system continued to monitor the activity of ambient insects.

The system detected very high numbers (> 1000/min) of ambient flying insects during the peak dusk activity period (Fig. 11). The release of 10,000 *A*. *aegypti* in the open section of the screen house at the peak dusk flight activity period did not produce a pronounced increase in insect detections, most likely due to the very high numbers of native crepuscular insects, that were active during the test period. Engagement of the laser during the initial 3 days of the test resulted in a pronounced decline in flying insect incidence relative to the fourth and subsequent days, when mosquitoes were released but the laser was not engaged (Fig. 11). When the laser was engaged during the first three nights of the test, the ratio of the baseline level of flying insects compared to the level of dusk-flying insects was 5% but when it was turned off during the remainder of the test, the ratio was 13%. The results showed that the system was capable of detecting and tracking enormous numbers of insects flying within the active zone.

### Flight elevation and speed observations

**Figure 12:**
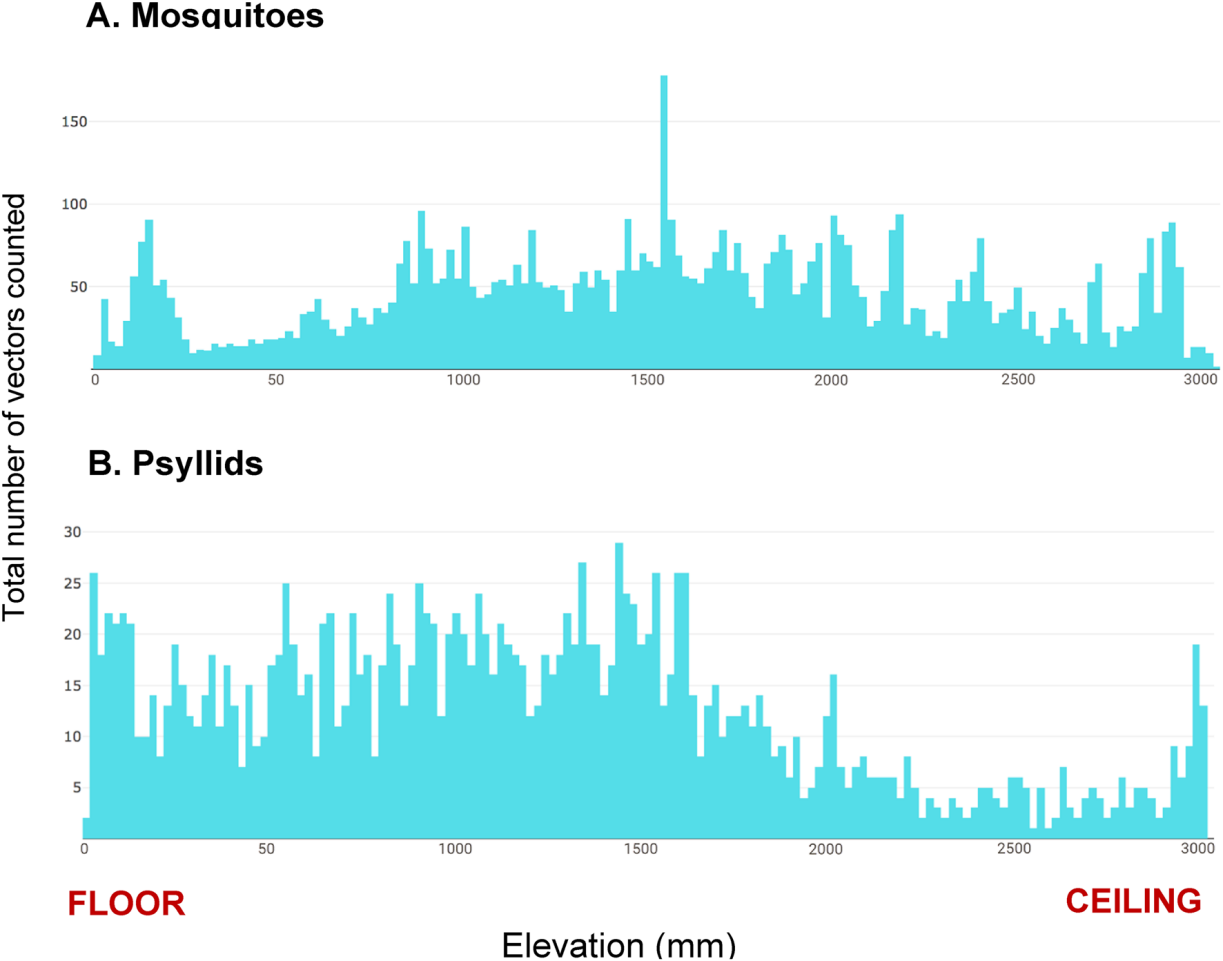
Flight elevation profiles of the flying insect vectors observed by the Photonic Fence. (**A**) Mosquitoes (*A*. *aegypti*); (**B**) Psyllids (*D*. *citri*).

**Figure 13:**
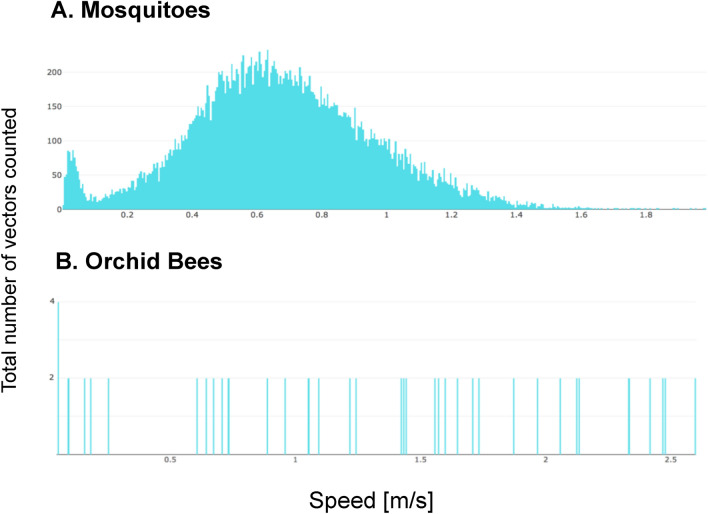
Speed profiles generated by the Photonic Fence of (**A**) mosquitoes (*A*. *aegypti*), showing a typical speed 0.4-1 m/s, and (**B**) orchid bees (*E*. *dilemma*), showing typical speeds of 0.5–2.5 m/s.

Each tracked insect trajectory through 3-dimentional space was timestamped and saved, along with the calculated size and sample target images from the fine tracking camera. The post-hoc evaluations of these flight data showed that the mosquito, *A*. aegypti, had a mean flight altitude of 1.3 m, which tapered off above 1.5 m. However, some individuals also flew near the screenhouse ceiling. (Fig. 12A). Asian citrus psyllids had no discernable flight altitude peak, but their flight tapered off at higher altitudes except for a peak close to the ceiling (Fig. 12B). Individual *A*. *aegypti* were observed flying at a typical velocity of 0.4–1 m/s (Fig. 13A) while individual male orchid bees (*E*. *dilemma*) were recorded flying at speeds ranging from 0.5 to 2.5 m/s (Fig. 13B).

## Discussion

During the initial test with 1000 *A*. *aegypti* (Fig. 5), the system registered an increase in mosquito activity when the human ‘agitator’ walked along the upwind side of the exterior of the screenhouse and a decrease in flight activity when the agitator walked away from the screenhouse (Fig. 6). When the agitator was next to the screenhouse, 300–400 mosquitoes per minute were detected but this number fell to below 100 per minute when the agitator walked away from the screenhouse. Because the agitator was located outside and upwind of the mosquitoes in the tunnel, and the screening would have obscured their visual appearance, the observed changes in mosquito flight responses observed during this test were likely due primarily to the concentration of olfactory cues emanating from the agitator which changed as the agitator moved along or away from the screenhouse^177^. This result demonstrated the system’s sensitivity to detecting and recording changes in localized vector activity. Once the laser was engaged, mosquito activity rapidly declined, proving the effectiveness of the lethal mode of the system. The results of this test also showed that the system successfully tracked mosquitoes throughout the active zone (Fig. 7), showing that there were no areas where detection and tracking was deficient and that up to seven individuals per second could be lethally engaged (Fig. 8).

In the tests of interception efficiency, only 0.9% of *A*. *aegypti* were recovered on the human subject side of the screenhouse at the completion of the laser-enabled test as compared to a 38% recovery in the non-lethal monitoring control (Table 1A), demonstrating that the Photonic Fence was capable of effectively interdicting large numbers of flying mosquitoes. The system was less effective at interdicting flying Asian citrus psyllid than mosquitoes (Table 1B). Because the system was designed to intercept targets the size of mosquitoes, tracking, targeting, and hitting the psyllid, which exhibits a significantly smaller detected cross-sectional area, proved to be challenging to the system (Fig. 10D). Improved efficiency with respect to intercepting smaller-sized targets may be achieved by modifying the system through measures that improve image resolution and the optical field-of-view, such as adding more powerful optical lenses, decreasing the area of the active zone, or adjusting the spot size of the laser beam. Tests with free-flying orchid bees showed that the system could distinguish target from non-target species (Fig. 10C). This is important because in most potential applications, the system must eradicate only vectors, not non-target or beneficial insects. The open-air test demonstrated that the system could track the high numbers of ambient flying insects that were active at dusk at the study site, with as many as 1500 insects per minute detected (Fig. 11). When the laser was engaged, decreases were observed in the numbers of insects detected by the system, showing that the laser could operate effectively, even when challenged with large numbers of potential targets.

This proof-of-concept Photonic Fence hardware architecture comprised several features optimized for performance characteristics and data logging. These attributes will be atrophied during the product design phase to optimize the commercial device for cost, size, and power consumption. Each Photonic Fence subsystem has been designed with an eye towards off-the-grid operation to enable deployment in locations without reliable power. The electrical power budget for the product version of the Photonic Fence is 200 W, making it operable with the use of commercially available solar panels or energy storage systems.

The results demonstrated that a field-scale sized Photonic Fence system could reliably and accurately detect, track, and identify hundreds of individuals of two different flying insect vector species and then kill them with a brief pulse of a low-power laser at a distance of up to 30 m. The Photonic Fence tested here effectively prevented flying vectors from infiltrating a 90 m^2^ active zone; and it was sensitive enough to accurately discriminate targeted from non-targeted insect species, intercepting and killing the vector species while leaving the non-targeted insects unharmed. The Photonic Fence represents a significant advancement to the overall development of laser-based insect vector eradication technologies^131–136^. Photonic exposure now represents a new mode of flying vector control with a very different mode of action: pin-point thermal damage to tissue in targeted individuals^127–129^ in contrast to insecticides, which work by disrupting neurological transmissions^178^. The application of topical insecticides to a targeted treatment area results in selection for individuals that can detoxify the active ingredient of the insecticide, which, in turn, drives insecticide resistance in that population^32,77,178^. The thermal damage inflicted by the laser pulse either kills the insect outright or severely incapacitates it, so individuals intercepted by the pulsed laser do not further contribute to the genetic composition of the population, leaving no possibility of ‘laser resistance’ developing in vector populations exposed to laser pulses. The thermal damage inflicted on the target affects only the target, and so it does not produce negative effects on people and communities, non-target organisms, and the local environment as does the persistent application of insecticides. Given these capabilities, there are many potential applications of the Photonic Fence and similar photonic devices in the interdiction, surveillance and investigations of flying insect vectors. These are discussed below.

### Applications of the Photonic Fence and similar photonic technologies

#### Vector interdiction

While the utility of the Photonic Fence specifically is discussed below, other types of optical and photonic systems, such as those mentioned above, could also potentially be used in these given situations. That the system was capable of intercepting large numbers of flying vectors throughout the 90 m^2^ active zone, demonstrated that the Photonic Fence could be used to establish ‘vector interception zones’ along the perimeters of areas in need of protection (Table 2). Strategic placement of vector interception zones would provide localized protection and help reduce insecticide reliance/resistance in vector populations at those locations. Further testing is needed to evaluate the efficacy and feasibility of the Photonic Fence in each of the application examples provided below.

**Table 2:** Examples of potential applications of the Photonic Fence to establish protective perimeters to detect, monitor, and intercept flying insect vectors at critical infection interfaces.

**Human health**
High at-risk villages & neighborhoods
Hospitals & health clinics
Schools, nurseries and playgrounds
Bus & train stations, markets, places of worship, and other gathering places
**Humanitarian crisis zones**
Refugee and displaced person shelters & camps
Forward hospitals and other health care facilities
Camps & housing areas for rescue & aid workers and peacekeeping personnel
Forward logistical and transportation hubs
**Livestock & animal health**
Barns, poultry houses, pens, and other rearing and holding areas
Truck trailers, railway cars, shipping containers
**Crop health & phytosanitation**
Quarantine & inspection stations, high-risk pest introduction areas at ports-of-entry and airports
Packing houses and distribution centers
Truck trailers, railway cars, shipping containers
Greenhouses, nurseries, and indoor growing areas
**Conservation & biodiversity**
Habitat fragments supporting endangered species
Zoos, botanical gardens & rearing facilities for endangered species
Areas supporting re-establishing endangered species

In terms of protecting human health, the Photonic Fence could be used to establish vector interception zones in health care facilities and other areas where people congregate, such as schools, places of worship, transit stations, or markets. This was the original intent and inspiration for the Photonic Fence^179^. As the technology improves and matures, Photonic Fences could be deployed outdoors, around the perimeters of buildings, villages and neighborhoods that are highly vulnerable to flying vector infiltration or to create a barrier between protected areas and high-density vector habitats, such as wetlands^180,181^.

Vector interception zones established around the perimeters of open areas will not result in the total exclusion of vectors in that area. However, because many vectors typically fly close to the ground while searching for a host^94,182–184^, a 3 m high Photonic Fence established around the perimeter of a protected area could be effective in reducing vector infiltration into protected areas. Significantly diminishing vector infiltration numbers is helpful because decreasing the numbers of resistant individuals in local populations improves the effectiveness of both localized treatments and regional insecticide resistance management programs^67,73,185^. Reducing vector infiltration would also help maintain the effectiveness of personal protection measures such as indoor residual spraying and insecticide-treated bed nets^186–189^. Establishment of vector interception zones around vector refugia, such as wetlands, would help reduce the numbers of vectors that disperse to nearby inhabited areas^180,181^. Because of the overlap in the insecticides used in both agricultural areas and human health protection, insecticide resistant vector populations from agricultural production areas threaten nearby human habitations^190,191^. Establishing vector interception zones in these areas will serve the dual purpose of decreasing vector populations while reducing the density of resistant individuals in the vector population. Because cattle and other livestock are alternate hosts for certain vectors that also feed on human blood^192–195^, establishing vector interception zones between areas where livestock are kept and human habitations could also be an important protection and control strategy.

Because refugees, migrants, and displaced people are very vulnerable to contagious and vector-borne diseases^196–200^, the Photonic Fence could be used to help reduce the spread of vector borne diseases in areas experiencing humanitarian crises due to natural disasters, climate change, or armed conflict. The establishment of vector interception zones could provide protection to people in refugee camps and shelters and at forward-based hospitals, transportation, and logistical centers. Providing flying vector interception zones would help prevent the spread of vector borne diseases under these dire circumstances.

Vector-borne diseases are an important problem agriculture, particularly in the care of domesticated animals^201^ and crop plants^20,202–204^. The Photonic Fence could be used to establish vector interception zones around or in agricultural production structures that are vulnerable to infiltration by flying vectors, such as livestock and poultry housing facilities^205–207^, greenhouses^208–210^ and produce packinghouses^211,212^. The Photonic Fence could also be used to provide surveillance in ports-of-entry, packinghouses and similar situations where pest detection and eradication is critical to preventing their release or spread^213,214^. It could be emplaced on conveyances such as truck trailers and railroad cars that transfer livestock and produce to prevent flying vectors from entering or exiting the cargo area. Further development of the Photonic Fence system will permit its deployment around open agricultural production areas such as orchards, vineyards, crop fields and pastures to reduce the level of flying vector infiltration into these areas^215–217^. The ability to establish protective active zones will be particularly helpful to combat exotic invasive vectors that have a high capacity to kill crops, such as the Asian citrus psyllid transmitting *Candidatus* Liberibacter species in citrus groves^151,152^. A similar situation occurs when native insects vector an introduced phytopathogen, such as native spittlebug species transmitting the causal agent of Olive Quick Decline Syndrome in southern European olive groves^218,219^. While the Photonic Fence will not totally exclude vectors from infiltrating the perimeters of outdoor areas, it will none-the-less reduce vector populations in the targeted area and can be used in combination with other control measures to provide more comprehensive control and reduce insecticide inputs.

The Photonic Fence could also be used to help protect endangered species and maintain biodiversity in targeted areas. For example, vector interception zones could be established around target areas supporting populations of at-risk species that are vulnerable to vector-borne diseases^220,221^, newly revegetated or reintroduction areas^222^, or reservoirs of vectors that negatively impact vulnerable species in nearby areas^223–227^. As with livestock protection, flying vector interception zones could be positioned in zoos and rearing facilities for endangered species^228–231^. Conversely, livestock and domestic animals harbor vector-borne diseases that can be transmitted to wildlife and native species, sometimes resulting in catastrophic consequences^232–234^. Establishing vector interception zones between areas where livestock and domesticated animals are reared and adjacent areas supporting wildlife could help decrease vector movement between these areas.

As conceived, field deployed units of the Photonic Fence will need about 200W to operate and can utilize solar panels or batteries as remote power sources. The Photonic Fence shuts-off if a large object entered the active zone, such as a worker or animal^129^. Ideally, the system would be set-up such that the field of view is not restricted, especially horizontally. This may restrict its use in situations where a clear field of view is not achievable across the desired dimensions of the active zone. Further refinements will enable the tracking system to disregard objects such as tree branches or poles in the field of view. To optimize the system for each vector species of interest, certain tracking algorithm parameters (e.g. detection threshold) or dosing laser conditions (e.g. power, spot size) will need to be altered^127–129^. A database with the flight parameters of several targeted vector species will need to be devised for a particular area. This would require establishing the flight parameters of each vector species expected in the targeted areas^129^. The system showed a high level of sensitivity with respect to vector v. bee detection and tracking, so it is expected be able to discriminate between target and non-target flying insects in applied situations. However, there is a need for further testing to document that the system can differentiate between friend and foe under ambient conditions. The Photonic Fence is designed to be autonomous, making it vulnerable to theft of its components and housing. To reduce theft and vandalism, efforts must be made to obtain the acceptance and participation of residents and stakeholders in implementing a Photonic Fence system in their locality^235–237^.

#### Vector surveillance

Access to real-time, accurate vector monitoring information within targeted areas, though often unavailable, is a critical component in vector-borne disease prevention and control^24^. As such, there is an urgent need for improved entomological surveillance capabilities, not only to provide effective routine vector and pathogen monitoring^238–241^, but to address the mounting challenges to effective vector control from insecticide resistance and the effects of climate change on the biology of vector-borne disease pathosystems^48,189,242–245^. By itself, the tracking system of the Photonic Fence is an effective means of monitoring flying insect vectors, one which has a practical operating distance, real-time identification of species of interest, and highly specific targeting and interdiction capabilities. Because the tracking modules can provide information about the incidence, movement, and distribution of insect species of interest in real time, they could be implemented as a stand-alone, high-quality entomological monitoring tool for vector surveillance as well as for fundamental and applied entomological research.

Most of the potential surveillance applications of the Photonic Fence overlap the interdiction scenarios presented in Table 1. For example, the expansion of global air travel and seaborne trade enables insect vectors to move great distances in short periods of time^11,246,247^. The Photonic Fence monitoring system could work alone or with other sensory systems to enhance vector surveillance and biosanitation in ports-of-entry, packinghouses and similar situations where vector detection is critical to preventing their release or spread^214,248^. The accurate, real time vector surveillance information provided by the Photonic Fence could help advance local and regional vector surveillance capabilities by improving early detection of the onset of vector population growth and dispersal, and signal that proactive measures need to be initiated or escalated to address impeding outbreaks^224,249^. It can also be used to augment meteorological, remote sensing, and other geospatial data, including from mobile devices, to identify and delineate key vector habitats and refugia, flight corridors, and areas vulnerable to vector infiltration within the targeted landscape. In addition to providing data on existing situations on the ground, this information can be used to improve predictions about the spatial and temporal distribution patterns of vectors in targeted landscapes^241,250–258^.

While not addressed in this study, devices that collect vector specimens intercepted by the laser can be incorporated into the system; these, in turn, could provide information about the prevalence and distribution of insecticide-resistant genotypes within vector populations in the target location^259,260^. This information would help inform the development and management of area-wide insecticide resistance management programs^244,261,262^ as well as help with epidemiological forecasting^263,264^. For example, the Photonic Fence could be used to identify where different types of vector resistance to available active ingredients occur and therefore where next-generation bed nets and insecticides would be most effectively used^244,265^. The monitoring capabilities of the Photonic Fence could be part of Integrated Vector Surveillance networks that utilize different monitoring approaches to provide comprehensive assessments of vector abundance and distribution in targeted areas and identify key vector interdiction points^266–268^.

The rapid detection system afforded by the Photonic Fence could also help facilitate identifying the different climate change drivers that influence vector abundance and distribution, providing improved forecasting of vector frequency and distribution under conditions imposed by climate change^45,269–271^. The system could also provide a variety of monitoring functions that would measure the incidence, movement, and distribution of insect species of interest, providing a valuable tool for ecological monitoring and conservation efforts aimed at endangered species and other species of interest^272,273^.

#### Flight Behavior

The Photonic Fence provided detailed 3-dimensional information about the flight characteristics of individual insects, demonstrating that it can be used as a tool to conduct studies of insect flight behavior. Each tracked insect trajectory through 3-dimentional space is timestamped and saved, along with the calculated size and sample target images from the fine tracking camera. This data is then available for post-processing by the user. In-depth knowledge about vector flight behavior is fundamental to developing a comprehensive understanding of vector ecology, behavior, and epidemiology^103,263^. The ability to readily conduct 3-dimensional tracking of vector flight will facilitate understanding key vector behaviors, such as their responses to living hosts, traps, insecticide-treated bed nets, and attractant and repellent substances, the behavioral underpinnings of host location as well as the efficacy of traps, attract and kill devices and ‘push–pull’ strategies, and, identifying flight entry points into dwellings or structures ^178,274–277^. This information, in turn, will facilitate evaluating and developing other surveillance and control measures such as traps, screening, insecticide-treated bed nets, and spray programs in dwellings and residential areas^278–285^.

The Photonic Fence presents a new optical means of accurately detecting, monitoring and controlling flying insect vectors in real time as well as providing new capabilities to investigate their flight behavior and mechanics. In terms of vector interdiction, the Photonic Fence can provide ‘vector interception zones’ to protect sensitive areas and augment current vector abatement measures. Further trials are needed to test the efficacy and flexibility of the Photonic Fence in the various scenarios described in this paper. Specific identification of vector species and gender will require a developing a database using collected vector flight and environmental data. Optimization of tracking and interdiction capabilities for each vector or gender will require modification of parameters such as tracking algorithms to improve optical resolution capability. However, once configured, the vector detection and tracking units of the Photonic Fence can be used alone to provide effective surveillance of sensitive areas. This may be extended to monitor beneficial insects and species of interest in a given situation.

## Methods and materials

### Study site

The study was conducted in a screenhouse (40 mL × 3.05 m W × 3.66 mH) at the USDA-Agricultural Research Service citrus grove in Fort Pierce, FL (Fig. 1A, B). The screenhouse was equipped with water lines and a 220v electric line to power the Photonic Fence.

### Insects

The eggs of the mosquito, *A. aegypti*, were obtained from colonies maintained by the USDA-ARS Laboratory in Gainesville, FL and from the University of Florida/IFAS Florida Medical Entomology Laboratory in Vero Beach, FL. The eggs and subsequent larvae were maintained in trays filled with RO water and supplied with standard rearing media containing dried liver and brewer’s yeast. Mosquitoes used in each test were from a single cohort. The rearing trays were kept in cages in an incubator set at 8 h L:16 h D photoperiod and held at 25 ± 1 *°*C temperature.

Adult Asian citrus psyllid (*D. citri*) were obtained from a research colony reared on orange jasmine (*Murraya paniculata*) seedlings and maintained in the controlled environment Insectary at the USDA Agricultural Research Laboratory in Fort Pierce, FL. The psyllid colonies were housed in screened cages containing a single potted orange jasmine seedling. The cages were kept on racks in the Insectary greenhouse and received supplemental lighting from an LED plant grow light. Both the immature and adult psyllids feed on the phloem sap of the orange jasmine seedlings. Only adults, 1–2-weeks post emergence in age, were used in the tests. Individuals for tests were collected 2–4 h prior the start of the tests and kept in ventilated plastic vials for transfer to the screenhouse. Both *A*. *aegypti* and *D*. *citri* are well-established at the test site in south Florida.

### Photonic fence set-up

The Photonic Fence was mounted on two aluminum towers that were anchored in the soil and placed 30 m apart from one another along the centerline of the screenhouse (Fig. 1A–C). The front-facing side of each tower was divided longitudinally into two parts: one half was covered by a reflective screen panel while the other side contained two Photonic Fence units, mounted on the top and bottom of the tower, and support equipment (communications system, laser generator, computers), mounted mid-tower (Fig. 2A). Each individual Photonic Fence unit consisted of four 850 nm illumination projectors, two coarse tracking cameras (100fps frame rate), and a single fine tracking camera (1,500fps frame rate) and laser system (Fig. 2B). The towers were mirror images of each other, so that the light beams projected from one tower were reflected from the reflector panel on the opposite tower. In total, four units were used to create the 30 m long × 3 m high × 0.3 m wide active space of the detection, interrogation, and interception system (Fig. 3). The reflector panels consisted of wide-angle, exposed retroreflective lenses bonded to a fabric substrate, attached to a metal frame (Fig. 2A).

The laser system was comprised of a continuous wave, randomly polarized fiber laser whose output was boresight-aligned with the fine tracking camera field of view. Once an insect was approved for engagement by the target validation algorithm and the steerable optic had centered on it, the laser was pulsed for a duration of 25 ms. The power delivered to the target was 25W, at a wavelength of 1550 nm. To assist with location and recovery of insects killed in flight, the gravel floor of the screenhouse was covered with white polyester fabric which provided a light background and contrast for locating the insects that had fallen there after interception by the laser pulse (Fig. 10D). In addition, clean room adhesive mats were placed on the ground immediately below the active zone, which provided both visual contrast and helped secure fallen insects. These areas were visually inspected after each test to count the numbers of fallen insects.

### Initial mosquito tracking and interception test

A 5.5-h long test was conducted to evaluate the capability of the Photonic Fence to detect, interrogate, and kill flying *A*. *aegypti*. At the start of the test, 1000 mosquitoes were released from three cages in the screenhouse. During the initial 4 h of the test, the detection and interrogation mode of the Photonic Fence was operational, but the laser was disengaged. To promote mosquito activity, a human ‘agitator’ walked slowly on the upwind side of the screenhouse (Fig. 5). One of the co-authors (AM) served as the agitator. All the methods were in accordance with relevant guidelines and regulations. After 30–45 min had passed, the agitator walked away from the screenhouse and then returned after a 15–20-min break to determine if the system could detect fluctuations in flight activity. During the last hour of the test, the laser was engaged to determine the system’s capability to intercept and kill flying mosquitoes while the agitator continued to walk outside. Post-hoc tests were performed to determine the system’s capability to detect flying mosquitoes across the active range of the system and the system’s laser engagement rate during the test.

### Flying vector interception efficiency tests

To determine the capability of the system to intercept flying mosquitoes and psyllids, tests were conducted in which the insects were released from cages on one side of the screenhouse. The cages were placed on the floor and were spaced equidistant from each other along the length of the active space of the system. Tests were conducted with the laser engaged or silent to compare recovery levels of released insects.

For tests with *A*. *aegypti*, 350 mosquitoes were released per test. Mosquitoes that flew across the active area of the Photonic Fence were recovered either from: (1) a pair of open pan oviposition traps; (2) a pair of home-made pail traps; or, (3) a single human subject (Fig. 9A). The human subject sat in a chair in the center of the screenhouse on the screenhouse side opposite of the release side (Fig. 9A). A pan trap and bucket trap were placed on the floor 5 M on either side of the human subject (Figs. 1C and 9A). This arrangement provided a total of three mosquito recovery points for the tests. During the tests, neither the human subject nor traps were in the line-of-sight of the Photonic Fence (Figs. 1C and 9A). The oviposition trap consisted of a 10L white plastic pan containing 6 L of infused water that had seeped in straw for 48 h^286^. The pail trap consisted of a 6 L bucket and lid and had design features similar to other autocidal gravid ovitraps^287–289^. For example, the exterior of the pail and lid was painted black, an attractive color to *Aedes*^290^. The housing of a miniature CDC trap (BioQuip), containing a UV LED lamp and downdraft fan, was inserted into a fitted hole cut into the lid and secured with hot glue. Two scent baits were placed inside the pail: a sachet containing a commercial mosquito bait (Sweetscent, Biogents AG) and ca. 500 mL of crushed dry ice held in an open thermos container^291^. When the fan was operating, it suctioned clean air into the bucket, and then air carrying the scent baits was exhausted through two screened holes (9 cm^2^) cut into the lid. Mosquitoes attracted to the trap were suctioned into its interior when they became caught in the fan downdraft. The human subject wore white coveralls and a mosquito-proof head net. A 100 cm^2^ patch of fabric was removed from the one leg of the coverall that exposed the subject’s thigh while seated. During the 60-min-long test, the subject used a manual aspirator to collect mosquitoes when they alighted on the patch of exposed skin^292^.

For tests with *D*. *citri*, 600 psyllids were released per test. Psyllids were recovered in 3-D yellow traps designed especially to attract and trap *D*. *citri* by the Florida Department of Plant Industry^293^ (Fig. 1D). The traps were manufactured with a 3-D printer and were bright yellow in the human visual spectrum, a color that is highly attractive to *D*. *citri*^294,296^. In addition, the surface features of the traps helped retain the psyllids and motivate them to enter the trap interior^293,297–299^. Psyllids that were caught in the trap slid downwards into a vial of soapy water. At the conclusion of the tests, the vials were returned to the lab and the psyllids examined under magnification for counting and sexing. Ten yellow 3D psyllid traps were used for each test. The traps were hung from a nylon line supported by 99 cm high aluminum fence posts (Tractor Supply Company) inserted into the gravel floor of the screenhouse. The traps were placed were positioned in the center of each screenhouse hoop panel and ca. 25 cm from the south facing wall of the screenhouse. Because the release cages were placed on the north-facing side of the screen house, the psyllids, which are phototrophic^297^, tended to move to the sun-lit, south-facing side of the screenhouse where the traps were positioned. The screen house panels were also examined for the presence of psyllids that were not captured by the traps. Psyllids found on the screens were included in the total number of recovered psyllids.

### Target discrimination test with male orchid bees

Male *Euglossa dilemma* were attracted to cotton swaps dipped in a scent bait, p-dimethoxybenzene^300^ (Sigma-Aldrich). The bees were collected with a net as they hovered near the scent bait (Fig. 4). The collections were made in a wooded residential neighborhood in Fort Pierce, FL, USA. The bees were collected in the morning, kept in individual plastic vials placed in a cool, dark storage box prior to the tests, and then used in the afternoon. At the start of the test, ten male E. *dilemma* were released in the screenhouse and allowed to fly freely for 60 min with the laser engaged.

### Open air test

To test the Photonic Fence’s capability to detect and interrogate ambient numbers of insects flying during the peak dusk activity period, a test was conducted with the screen removed from the ten center support hoops of the screenhouse (Fig. 1E). This allowed native insects from the surrounding environment to fly across the active space of the Photonic Fence. The test was conducted over a 10-day period. The laser was engaged from the start of the test until 96 h had elapsed. During the initial 96-h period, three releases were made, 10 m upwind of the screenhouse, of 10,000 *A*. *aegypti* at the peak period of dusk flying activity (approximately 18:00 h Eastern Daylight Time). This test was conducted to determine (a) if a very large number of vectors could be targeted by the system when ambient insect levels were also very high, and (b) the natural flight elevation distribution of *A. aegypti*. A control test was conducted at dusk on day 5 in which 10,000 mosquitoes were released but the laser remained silenced. For the remaining 4 days, only monitoring and tracking of ambient insect levels was recorded.

## Data Availability

The datasets used and/or analyzed during the current study available from the corresponding author on reasonable request.
